# Green solvent mediated extraction of micro- and nano-plastic particles from water

**DOI:** 10.1038/s41598-023-37490-6

**Published:** 2023-06-30

**Authors:** Jameson R. Hunter, Qi Qiao, Yuxuan Zhang, Qing Shao, Czarena Crofcheck, Jian Shi

**Affiliations:** 1 Biosystems and Agricultural Engineering, University of Kentucky, Lexington, KY 40506 USA; 2 Department of Chemical and Materials Engineering, University of Kentucky, Lexington, KY 40506 USA

**Keywords:** Chemical engineering, Sustainability

## Abstract

The production of plastic and the amount of waste plastic that enters the ecosystem increases every year. Synthetic plastics gradually break down into particles on the micro- and nano-scale in the environment. The micro- and nano-plastics pose a significant ecological threat by transporting toxic chemicals and causing inflammation and cellular damage when ingested; however, removal of those particles from water is challenging using conventional separation methods. Deep eutectic solvents (DES), a new class of solvents composed of hydrogen bond donors and acceptors, have been proposed as a cheaper alternative to ionic liquids. Hydrophobic DES derived from natural compounds (NADES) show promise as extractants in liquid–liquid extractions. This study investigated the extraction efficiency of micro- and nano-plastics including polyethylene terephthalate, polystyrene, and a bioplastic polylactic acid from fresh water and saltwater using three hydrophobic NADES. The extraction efficiencies fall in a range of 50–93% (maximum % extraction) while the extraction rates fall between 0.2 and 1.3 h (as indicated by the time to extract half the theoretical maximum). Molecular simulations show a correlation between the extraction efficiency and the association between the plastics and NADES molecules. This study demonstrates the potential of hydrophobic NADES as extractants for removal of different micro- and nano-plastic particles from aqueous solutions.

## Introduction

Plastics have become a vital part of daily life for both consumers and industries due to their strength, stability, durability, and low synthesis cost^1^. However, the plastics produced on a massive scale are typically resistant to environmental degradation, causing plastic waste to accumulate in landfills and act as a major pollutant in the ocean^2–6^. Plastic waste in a marine environment can be divided into three categories, depending on its size: macro-plastic (> 5 mm), micro-plastic (1000 nm–5 mm), and nano-plastic (< 1000 nm). Nanoplastics could be generated by various sources^7,8^, ranging from mechanical stress to chemical degradation. Large pieces of plastic in the marine environment slowly break apart due to UV degradations and buffeting waves^3,4^. When ingested, micro- and nano-plastics have been reported to cause inflammation and even damage at the cellular level^9^. Due to the hydrophobic surface of plastic particles, they also have the potential to adsorb and concentrate toxic hydrophobic substances that are already present pollutants in the environment and transport them over long distances through ocean currents^10,11^.

Two of the six most common petroleum-based plastics produced are polyethylene terephthalate (PET) and polystyrene (PS), accounting for 10% and 8% of the global plastic production, respectively^12,13^. These plastics in their micro- and nano particulate forms are commonly found in the marine environment^14^. Polylactic acid (PLA) is a relatively new alternative made from renewable resources that is designed to degrade faster than petroleum-based plastics and will likely become more common in coming years^15^. PLA has been shown to decompose easily under thermophilic conditions such as industrial composting and high temperature anaerobic digestion^16–18^; however, its degradability in mesophilic environments such as ocean and soil is compromised and resulted in partial degradation or PLA fragments^19–21^.

While strategies exist to prevent plastics from getting into the environment, there is a vital need to also have strategies for plastic removal from the environment. Recovery of waste plastics will also open possibilities to recycle and reuse the materials^22–24^. Currently employed recovery and separation techniques for micro- and nano-plastics are generally ineffective, time-consuming, or specific only to certain particle sizes^9^. Passive density and size separation methods such as centrifugation, filtration, and sedimentation, are not well-suited for particles on the micro- or nano-scale^25–27^, due to the their different properties, such as buoyancy and surface charge from macro-plastic particles, making these separation techniques less effective^25^. Neuston net and coring process have been used to extract microplastics for analytical studies, but they are only used to collect small amount of samples and would have issues scaling up for plastic recovery^9,28^. Once recovered from the environment, those nano- and microplastic particles can be potentially reused or valorized into other products via thermochemical or biological upcycling methods^29,30^.

Deep eutectic solvents (DES) are a class of recently discovered solvents formed by a combination of hydrogen bond donors and acceptors^31,32^. Typically, the individual components are a solid at room temperature, but when mixed have a melting point much lower than either component. DES have low volatility, a wide liquid range, are water-compatible, are non-flammable, and they are typically biocompatible. Two of the largest advantages of DES over ionic liquids are in the lower cost of components and the ease of preparation^31,33^. The first DES reported was composed of amide and choline chloride, where the components liquified upon contact, most likely due to hydrogen bonding and van der Waals interactions between the components^34–36^. In recent years, there has been a push for solvents that are less toxic and are made from natural materials. A sub-category of DES called natural deep eutectic solvents (NADES) are made from non-toxic components derived from natural materials, like the monocyclic monoterpenoids menthol and thymol, organic acids, and salts^34,37^. These NADES are generally accepted as environmentally friendly and have the potential to obey the 12 Principles of Green Chemistry set forth by Anastas and Warner^38^.

Hydrophobic NADES is a promising candidate for extracting organic substances from the aqueous solutions because they are relatively cheap and less toxic as compared to the other common solvents^37,39–48^. However, to the best of our knowledge, hydrophobic NADES has not been investigated for micro- and nano-plastics extraction. The objectives of this work are to synthesize and characterize the hydrophobic NADES, develop a method for synthesizing and characterizing plastic nanoparticles, and determine extraction efficiency and rate of plastic particles by the hydrophobic NADES in both freshwater and saltwater. Molecule dynamic simulations were carried out to study molecular interactions between plastic and NADES molecules and correlations to the extraction efficiency. Collectively, this study demonstrates the potential use of NADES as extractants for removal of different micro- and nano-plastic particles from aqueous solution and helps to guide the design of hydrophobic NADES and the development of processes to remediate plastics from aqueous systems.

## Materials and methods

### Materials

The materials for synthesizing NADES including decanoic acid (≥ 98% purity), menthol (≥ 99% purity), and thymol (≥ 99% purity), and the granular PET were purchased from Sigma-Aldrich. PS pellets were purchased from Acros Organics. Granular PLA, films of PET and PS were purchased from GoodFellow Corporation (Coraopolis, PA). Chemicals used in the preparation of the plastic particles: phenol, *p*-xylene, and ethanol (all > 99% purity), were all purchased from Sigma-Aldrich, while acetonitrile (> 99% purity) was purchased from VWR.

### NADES synthesis

NADES were synthesized by combining two components according to the given molar ratios. For this study, a 1:1 and 1:2 molar ratio of decanoic acid:menthol and a 1:1 molar ratio of thymol:menthol were used^34^. The mixtures were placed into capped glass bottle and heated at 40 °C with periodic vortexing until forming a clear liquid. The synthesized NADES were kept in capped bottles in a desiccator at room temperature until use.

### Contact angle measurements

The contact angle of NADES on plastic films were tested using a KRÜSS DSA100S Drop Shape Analyzer (tensiometer, KRÜSS Scientific Instruments, Inc., Matthews, NC). PET and PS films were commercially available, while the PLA film was synthesized in the lab by spreading a solution of 20 wt% PLA in acetonitrile on a sheet of clean glass into a thin layer of 200 μm thickness using a doctor blade casting unit. The film was left under a fume hood allowing the acetonitrile to evaporate. The film was then soaked in DI water overnight, wiped with paper towels and allowed to air dry.

To measure, 3 μL of NADES was dropped on a 4 cm^2^ plastic film secured to a glass microscope slide. The syringe was lowered until the tip of the needle is visible in the camera and the plunger was pushed so a droplet formed at the tip. Resolution was adjusted to focus the droplet and the arm was lowered until contact is made with the film. The needle was raised, and contact angle measurements were taken at 0 s and every 10 s for the next minute. Each NADES was measured twice on each plastic film, with the syringe being washed with ethanol between changes in NADES.

### Plastic particle preparation

PET particles were prepared by solubilizing ground granular PET in phenol and then precipitating in ethanol. In brief, 0.1 g of PET was dissolved into 4 g of phenol in a capped glass bottle on a magnetic stir plate set at 80 °C and 250 rpm for 2 h (any insoluble particles were discarded). Using a glass Pasteur pipette, the phenol/PET mixture was slowly dripped into a 100 mL beaker with 50 mL of ethanol being stirred at 400 rpm using a magnetic stir bar. The mixture was centrifuged at 4000 rpm for 10 min and the particles were washed with 40 mL of DI water for 3 times. The final concentration was adjusted to 1 mg/mL of PET in DI water. The mixture was stored at room temperature in a lidded glass container. The PS and PLA particles were prepared like the PET particles except that ethyl acetate and acetonitrile were used as solvents for PS and PLA, respectively.

### Particle size distribution, zeta potential, and imaging

Particle size distribution and zeta-potential were measured using dynamic light scattering (DLS) on a Malvern Panalytic Zetasizer Nano ZS (Westborough, MA). The plastic particle samples were diluted to 0.05 mg/mL in water and saltwater (3.5% NaCl) and vortexed before reading. Each sample was measured three times.

The synthesized plastic particles were imaged using a scanning electron microscopy (SEM). The freeze-dried samples were attached to a mount then sputter-coated with gold to enhance sample conductivity. Images were obtained at beam accelerating voltages of 2 kV using a FEI Quanta 250 FEG SEM operating at SE mode under low vacuum (0.40–0.65 Torr).

### Plastic particle extraction using NADES

Extraction experiments were conducted by introducing 2 mL NADES (top phase) to 2 mL plastics containing aqueous phase (bottom phase) in a glass vial at room temperature. The bottom phase was stirred using an 8 mm diameter SpinFin stir bar at 500 rpm. The initial plastics concentration of the aqueous phase was 1 mg/mL plastics in DI water. A 40 μL sample was taken from the bottom phase at predetermined time points. The turbidity of the sample was measured at 400 nm optical density (OD) on a SpectroMax M2 spectrophotometer. The OD readings were converted to concentration using calibration curves created using a set of known samples containing 0.05, 0.1, 0.2, 0.5, 0.8, and 1 mg/mL of plastic particles, respectively. For PET, the extraction lasted 32 h. PS extraction was conducted similarly to the PET extraction, but the timeframe was extended to 32–96 h due to slow extraction rate. For PLA extractions, the timeframe was reduced to 5 h due to fast extraction rate.

Extraction efficiency (% extracted) was calculated by comparing the measured concentration to the initial concentration. Regression analysis of the extraction efficiency over time was done using single rectangular curve fitting^49^. The model can be applied to the percent of plastic particles extracted over a period of time with the equation:

$$y=a\frac{x}{b+x}$$

In this equation, $y$ represents the percent of plastic particles extracted, $x$ the time since the start of extraction, $a$ the theoretical maximum of particles extracted from the aqueous phase, and $b$ the time point where half of the theoretical maximum particles have been extracted from the aqueous phase. This model is functionally identical to the Langmuir adsorption isotherm to describe adsorption on a particles surface.

### Molecular dynamics simulations

Interactions between the plastic molecules and NADES/water were studied using molecular simulations. The plastic molecules and NADES were described using the all-atom model and water molecules were described using the TIP 4P model^50^. Bonded and nonbonded interactions within the system were determined using OPLSAA/M force field^51^ which can describe the behavior of organic molecules, and its parameters were assigned using the Ligpargen web server^52–54^. The simulation systems were created within a cubic box with one plastic molecule and a specific number of solvent molecules. The simulation program was GROMACS and the system was created using the insert-molecule and solvate tools^55^. The detailed modeling parameters and settings can be found in ESI.

### Statistical analysis and regression

Statistical analysis was conducted using the data analysis tool in Microsoft Excel while regression analysis of extraction data was conducted in SigmaPlot 14.0. An analysis of variance (ANOVA) with a significance level of *p*-value less than 0.05 were conducted with the null hypothesis that all the means were equal. In the case where the null hypothesis is rejected, two-sample, two-tail t-tests assuming unequal variances were conducted between each mean to determine which means are significantly different from each other.

## Results and discussion

### Plastic particle synthesis and characterization

Characterization of the synthesized plastic particles is necessary because the size and shape of the material will have an impact on the extraction rate and efficiency. Smaller particles have lower individual surface area and tend to form more stable colloid systems which lead to a decrease in the extraction rate and efficiency.

**Figure 1:**
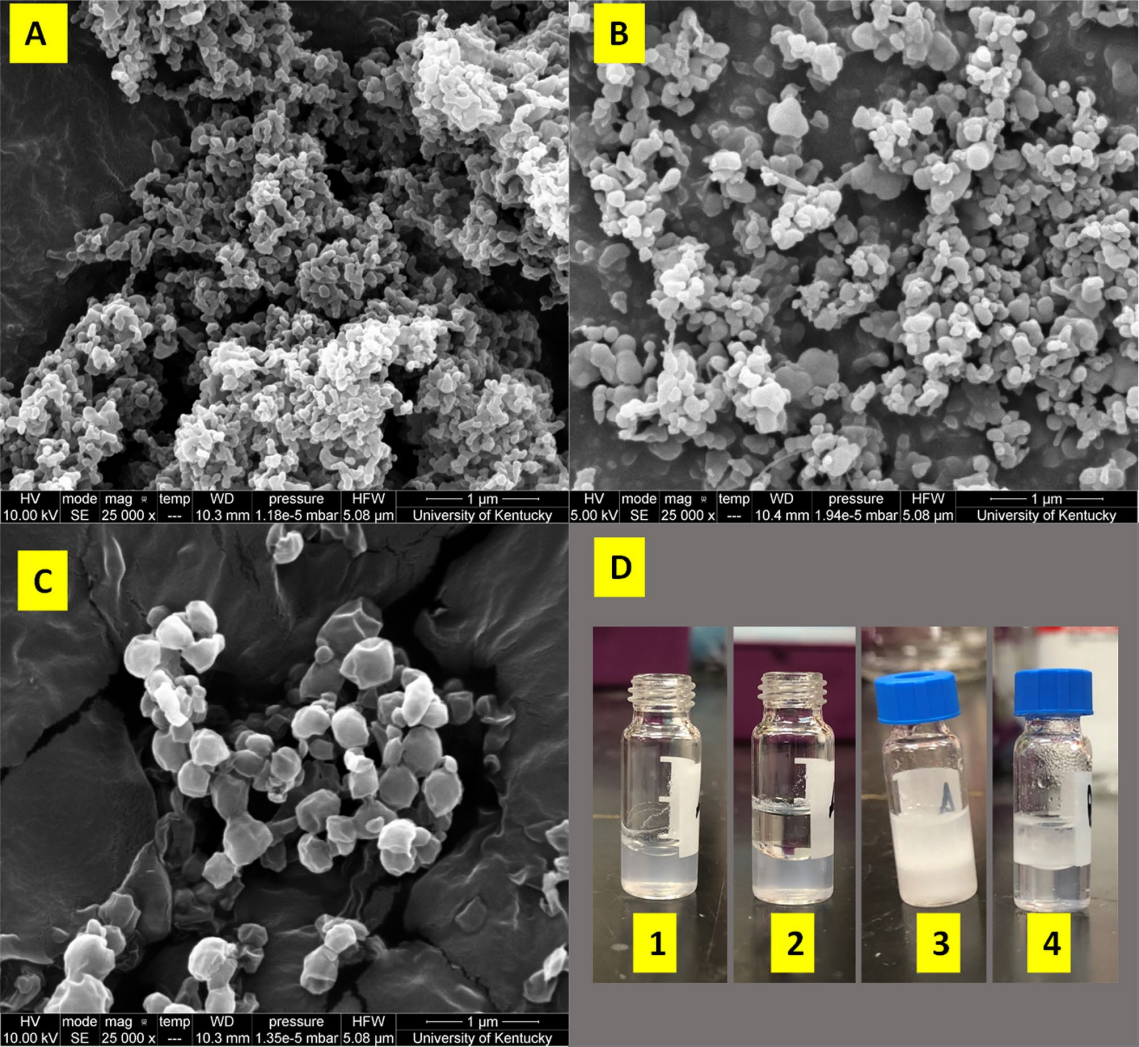
Schematic overview of the NADES extraction of plastics particles. Scanning electron microscopy images of (**a**) PET, (**b**) PS, and (**c**) PLA particles; (**d**) Hydrophobic NADES extracting microplastic particles from aqueous solution. In Fig. 1d from left to right: (1) PET plastic particles in aqueous solution, (2) NADES (top phase) and PET plastic particles in aqueous solution (1:1 v/v) before mixing, (3) mixture of NADES (decanoic acid: menthol = 1:1) with PET plastic particles in aqueous solution (1:1 v/v) right after mixing, and (4) the PET plastic particles migrated to NADES after phase separation.

The shape and size of the synthesized plastic particles in DI water were characterized using SEM and DLS, respectively (Fig. 1 and Table 1). The plastic particles appeared to be spherical, or rod shaped with varying particle sizes depending on the type of plastics. Z-average is a measurement of cumulant size within a sample and polydispersity index (PDI) measures how disperse the particles are within the media; the closer to zero the PDI is the more uniform a sample is. All synthesized plastic particles had an average size less than 1000 nm in water with relatively small PDI, classifying them as nano- or micro-plastics for this study. Since the average particle size is below 1 micron, the synthesized particles are in the submicron range as compared to the large microplastic particles. PET and PS particles were generally smaller, as compared to PLA particles.

**Table 1:** Particle size and zeta-potential of plastic particles in water and saltwater (n = 3).

	Z-average ± SE (nm)	PDI ± SE	Zeta potential ± SE (mV)
PET in water	334 ± 3	0.234 ± 0.016	− 24.7 ± 0.28
PS in water	263 ± 1	0.199 ± 0.015	− 29.4 ± 1.37
PLA in water	775 ± 16	0.464 ± 0.034	− 19.3 ± 1.23
PET in saltwater	3870 ± 476	0.923 ± 0.077	− 10.9 ± 3.04
PS in saltwater	3087 ± 254	0.351 ± 0.061	− 15.8 ± 1.67
PLA in saltwater	5769 ± 443	0.335 ± 0.027	7.01 ± 1.40

The sizes of the plastic particles in 3.5% sodium chloride (saltwater), in a range of 3000–6000 nm were much larger than their sizes in DI water, likely due to a change in the zeta potential thus causing aggregation in the saltwater solution. Nevertheless, the average sizes of PET and PS particles were smaller than the PLA particles in saltwater water, but the PET particles became less uniform than the other particles as indicated by the high PDI.

The liquid layer surrounding a particle exists as an inner layer with strongly bonded ions and a diffuse layer further away where the particle is less firmly associated. Between these layers is a slipping plane and the potential across it is the zeta potential. The DLVO theory suggests that colloid stability is dependent on the total potential energy of a particle^56,57^. The two majorly contributing forces are the van der Waals attractive and electric double layer repulsive forces. The equation to describe the van der Waals attractive force is ${V}_{A}=\frac{-A}{12\Pi {D}^{2}}$ where $A$ is the Hamaker constant, $\Pi$ is the solvent permeability, and $D$ is the particle separation. The equation for the electric double layer repulsive forces is ${V}_{R}=2\Pi a{\zeta }^{2}{e}^{-\kappa D}$ where $a$ is the particle radius, $\zeta$ is the zeta potential, and $\kappa$ is a function of the ionic composition. If the repulsive forces are greater than the attractive forces, the particles will stay separated and form a colloidal system. As a well-recognized index for assessing the stability of a colloid system, zeta potentials over ± 30 mV are considered stable, values between ± 15–30 mV being moderate stable, and values between ± 0–15 mV are prone to aggregate (not stable)^58^.

Zeta potentials of the three plastic particles in water spanned in a range of − 20 to − 30 mV. In saltwater, zeta potential of the three plastic particles became less negative (− 10 to − 15 mV for PET and PS) or slightly positive (7 mV) for PLA. As the zeta potential gets closer to zero, the repulsive forces decreases and once the attractive forces are larger, the suspension becomes unstable, and particles aggregate. It has been documented in previous studies that the addition of sodium chloride to a solution will decrease the negativity of zeta potential around particles. Prathapan et al. (2016) reported a less negative zeta potential of cellulosic nanocrystals in increasing concentrations of sodium chloride^58^. The introduction of sodium ions encourages adsorption to the surface of the particles and compresses the size of the double layer. In some cases, the adsorption of ions can even reverse the charge on the surface, which was observed in the case of PLA in the sodium chloride solution.

From the equation for repulsive forces, both particle size and zeta potential have an influence on the stability of suspension of particles. However, if the particles are too large, the inertial and gravitational forces will have a larger impact than the attractive and repulsive forces and prevent a colloid from forming. The plastic particles in saltwater indeed start to aggregate and precipitate after 24 h of storage. Therefore, in this study, we focused on extraction of plastic particles from fresh water.

### Contact angle analysis

**Figure 2:**
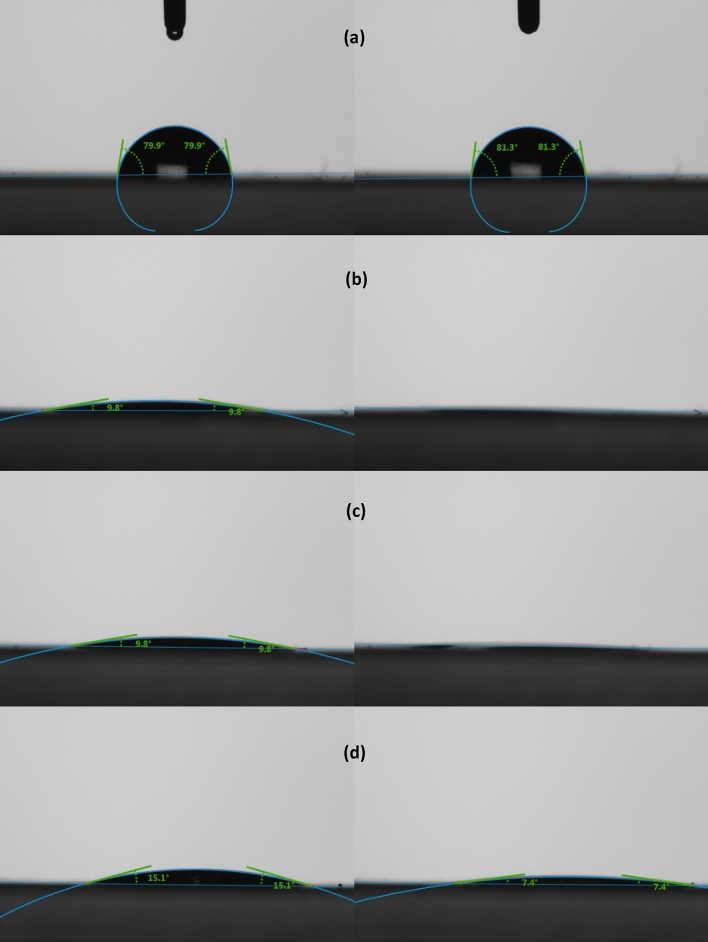
Contact angles of (**a**) water, (**b**) decanoic acid:menthol (1:1), (**c**) decanoic acid:menthol (1:2), and (**d**) thymol:menthol (1:1) at 0 s (left) and 30 s (right) on PET surface.

The water-repellent property of plastics and the affinity between plastics and DESs were characterized by measuring the static contact angle, which is determined between a liquid NADES droplet and a plastic surface. This test was conducted to determine the affinity between the plastic particles and the NADES and water. The hydrophobic nature of the NADES creates an interface layer between the NADES and water in the extraction system. Because the plastics particles tested are hydrophobic, they are more likely to move into the hydrophobic phase to when it encounters the interface layer during extraction. The contact angles of the three NADES on plastic films were compared with the contact angle of water droplet on plastic films (Fig. 2 and Figure S1 and S2). Overall, all three plastic films exhibit hydrophobic surfaces as showed by the relatively large contact angles (near 90°) with water droplet. However, the NADES droplets on the plastic films showed a contact angle much less than 90°, suggesting a strong affinity between plastic and NADES. For the PET and PS films, the NADES would spread upon contact with films. This can be seen in the decreasing contact angles between the initial measurement at 0 s and a later measurement at 30 s. For the PET film, the two decanoic acid:menthol NADES showed a similar affinity, while the thymol:menthol (1:1) had slightly higher contact angles. On the PS film, the decanoic acid:menthol (1:2) NADES showed the strongest affinity (smaller contact angle), while decanoic acid:menthol (1:1) and thymol:menthol (1:1) had similar results. Between the two plastics, each of the NADES had a smaller contact angle on the PET film than they did on the PS. The contact angles of NADES on the PLA film (even though < 30°) were higher than the those on the other two films. This is possibly caused by the chemical structure of PLA containing more hydrophilic carboxyl groups. But these could also be attributed to a rougher surface or abnormalities in the thickness of the film as it was synthesized in the lab using a casting method rather than the calendar rolling in a commercial manufacturing process.

The contact angle a liquid makes on a surface is a measurement of its wettability. Wetting measures the ability of a liquid to make and maintain contact with the surface of a solid and is influenced by intermolecular forces such as adhesion between the liquid and the solid and the cohesion within the liquid itself. Lower contact angles correlate to a higher wettability and extremely high wettability results in the spread of a liquid across the surface instead of forming a droplet. The NADES showed great wettability on the commercially manufactured plastic films. On the PET film, there was no statistically significant difference between the initial contact angle for the two decanoic acid:menthol NADES mixtures, but for thymol:menthol (1:1) the initial contact angle was significantly higher. After contact, each of the NADES spread reaching angles lower than five, which is the minimum angle the instrument can measure, in all measurements but one thymol:menthol (1:1) and no significant difference was found. The PS film showed higher initial contact angles with no statistically significant difference between the NADES. After 30 s, the NADES spread with decanoic acid:menthol (1:1) being significantly higher and no significant difference between the other two NADES. Between the two films, the decanoic acid:menthol (1:1) NADES had an initial contact angle significantly higher on PS than PET, but there was no significant difference between the angles after 30 s. Decanoic acid:menthol (1:2) NADES also had significantly different initial contact angles with PS being higher than PET, and by 30 s both had reached contact angles less than 5°. Thymol:menthol (1:1) NADES showed no significant difference in the initial contact angle or contact angle after 30 s. For PLA film, the contact angle results are less consistent than the commercially manufactured PET and PS films. However, the difference between the water and NADES are different to the point that a conclusion can be made that the NADES have more wettability on PLA than water.

### Plastic particle extraction using NADES

**Figure 3:**
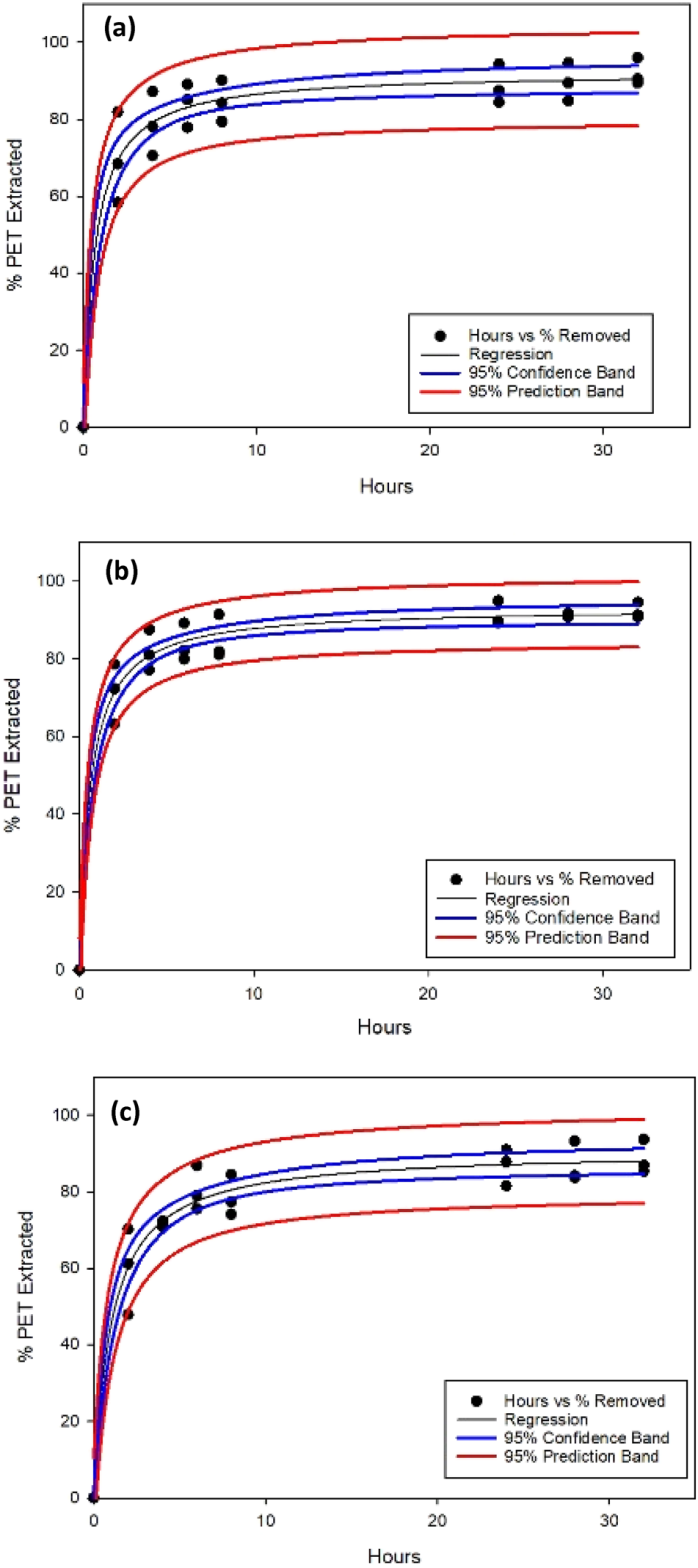
Percent of PET extracted by three types of NADES: (**a**) Decanoic Acid:Menthol (1:1); (**b**) Decanoic Acid:Menthol (1:2); and (**c**) Thymol:Menthol (1:1).

**Figure 4:**
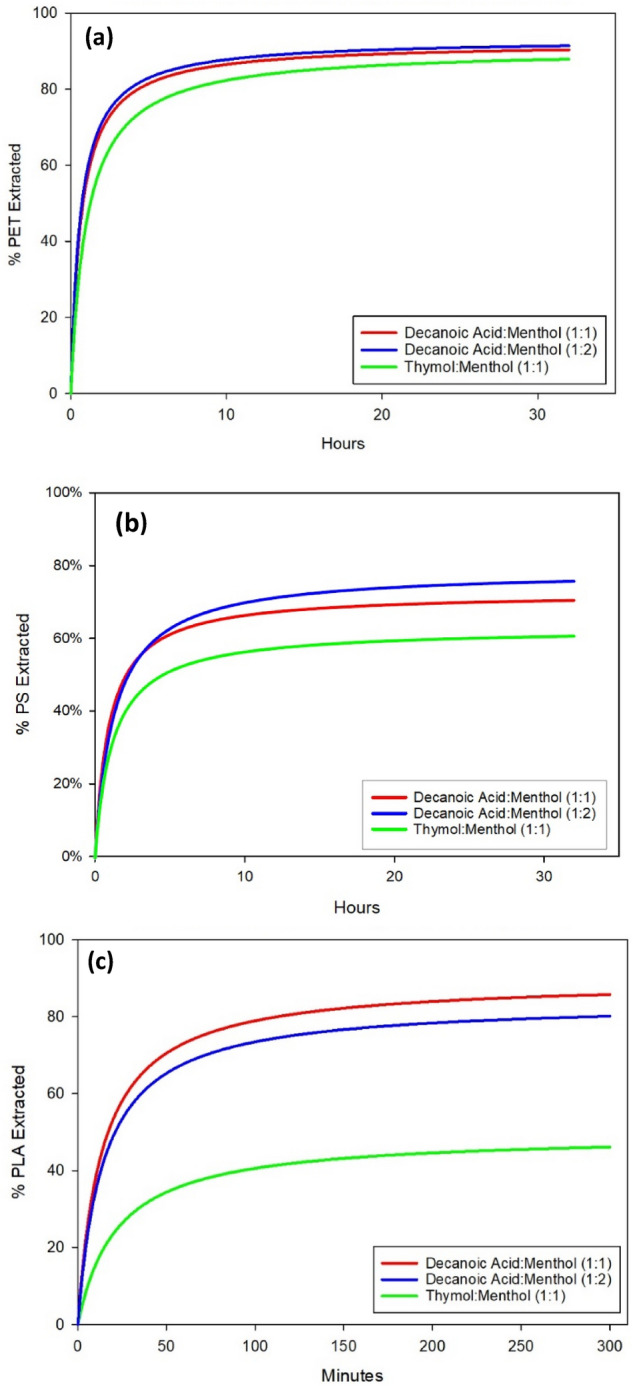
Summary of the extraction efficiency of (**a**) PET, (**b**) PS, and (**c**) PLA by three types of NADES.

The performance of NADES on extracting plastic particles can be determined by the extraction rate and overall extraction efficiency. In order to compare across different NADES and plastics, we conducted extraction experiments under well controlled conditions (the same temperature, volume ratio, and stirring speed). In such a setup, the aqueous phase is in contact with the NADES phase through an interface rather than complete mixing (like vertexing). The extraction rate is lower; however, it allows for comparison among different plastic-NADES combinations. The extraction of PET and PS particles reached equilibrium in around 18–24 h (Figs. 3 and 4). The overall extraction efficiency for PET is around 80–90% while in comparing the three NADES, decanoic acid:menthol (at 1:1 and 1:2 ratios) showed a slightly better extraction performance than that of thymol:menthol (1:1). The extraction efficiency of PS particles was generally lower than PET extraction, falling in the range of 55–75%. Decanoic acid:menthol NADES (at 1:1 and 1:2 ratios) outperformed thymol:menthol (1:1) NADES. The extraction of PLA particles took place over a 5-h period due to a significantly higher extraction rate than the other two plastic particles. Again, NADES made of decanoic acid:menthol combinations (1:1) showed ~ 80% extraction efficiency which were much greater than the ~ 40% extraction efficiency by NADES made of thymol:menthol (1:1).

Measurements taken during the extraction of plastic particles from salt-water proved to be inconsistent. The aggregation of the particles made it difficult to get an accurate representation of concentration with the relatively small sampling size in this study. These challenges made it impossible to create a curve for rate of extraction, however, visual observations of extraction in salt-water suggest a significantly faster extraction. The aggregation of particles makes them more visible. For each plastic type, at the same concentrations as the freshwater experiments, most visible particles had transferred to the NADES phase after 10–20 min. It has been documented that the rate of extraction can be influenced by both the size of the particles and the zeta potential of the system^9,25^. Given the significantly larger plastic particle sizes (3000–6000 nm) and their closer to zero zeta potential (− 16 to 7 mV) in saltwater, it is evident that separation/extraction of plastics particles from saltwater is comparably easier than separating them from fresh water.

**Table 2:** Contact angles for water and NADES on the plastic films.

	Initial contact angle ± SE			Contact angle after 30 s ± SE		
	PET	PS	PLA	PET	PS	PLA
Water	79.9	88.3	92.07	81.3	87.7	90.23
Decanoic Acid:Menthol (1:1)	9.92 ± 0.17	24.01 ± 2.72	27.85 ± 7.56	< 5	11.32 ± 2.14	21.43 ± 0.93
Decanoic Acid:Menthol (1:2)	10.85 ± 1.49	15.48 ± 0.03	29.23 ± 1.22	< 5	< 5	23.53 ± 0.50
Thymol:Menthol (1:1)	14.38 ± 0.95	21.89 ± 3.15	28.85 ± 1.25	< 5	7.38 ± 0.60	28.68 ± 1.27

Contact angle of < 5 degree is below the detection limit therefore no standard error is reported; n = 3 for the averages.

In order to compare across the NADES and plastics, we conducted regression analysis of the extraction efficiency over time using single rectangular curve fitting^49^. The fitted equation contains two coefficients: $a$ representing the theoretical maximum of particles extracted from the aqueous phase, and $b$ representing the time point (in hours) where half of the theoretical maximum particles have been extracted from the aqueous phase (Table 3).

**Table 3:** Comparison of the regression coefficient of plastics extraction by three types of NADES, where α represents the theoretical maximum of particles extracted from the aqueous phase, and β represents the time point where half of the theoretical maximum particles have been extracted from the aqueous phase.

NADES	PET		PS		PLA	
	α	β	α	β	α	β
Decanoic Acid:Menthol (1:1)	92.21	0.66	72.78	0.93	89.65	0.226
Decanoic Acid:Menthol (1:2)	93.19	0.62	79.04	1.29	83.95	0.238
Thymol:Menthol (1:1)	90.71	1.02	62.58	1.12	49.48	0.365

For PET extraction, the two decanoic acid:menthol NADES performed similarly in terms of the maximum extraction efficiency ($a$) and half time $b$. Thymol:menthol (1:1) NADES showed slightly lower maximum extraction efficiency and slower extraction rate as indicated by the larger $b$. All three NADES had theoretical maximums of at least 90% and extracted half of that maximum within or just past one hour of extraction. For PS extraction, the three NADES had varying performances. According to the regression, decanoic acid:menthol (1:2) had the highest extraction efficiency at 79%, followed by decanoic acid:menthol (1:1) at 72% and thymol:menthol (1:1) at 62%. In term of extraction rates, all three NADES need similar time (0.93–1.29 h) to reach half of their maximum extraction. For PLA extraction, the decanoic acid:menthol NADES (at 1:1 or 1:2 ratio) performed similarly well. Thymol:menthol (1:1) had a much lower maximum extraction of less than 50% and a slightly slower extraction rate reaching half its maximum extraction. However, for all PLA extraction, the extraction rates were much higher than extraction of the PET and PS.

### Molecular simulations

Interactions between the plastic and NADES molecules within the simulation were measured by their radial distribution function (RDF), which describes the probability of finding a particle a given distance from a reference particle. A higher RDF indicates that the particle is more likely to associate with the reference particle. Figure S5 shows the reference oxygen, hydrogen and carbon atoms that used to calculate RDF. For PET and PLA, the chosen particles were the oxygen atoms, and the reference particles were the hydrogen atoms of hydroxyl groups on decanoic acid, menthol, and thymol. PS does not contain oxygen, so the four carbon atoms on the mainchain were used with the hydrogens on the NADES.

**Figure 5:**
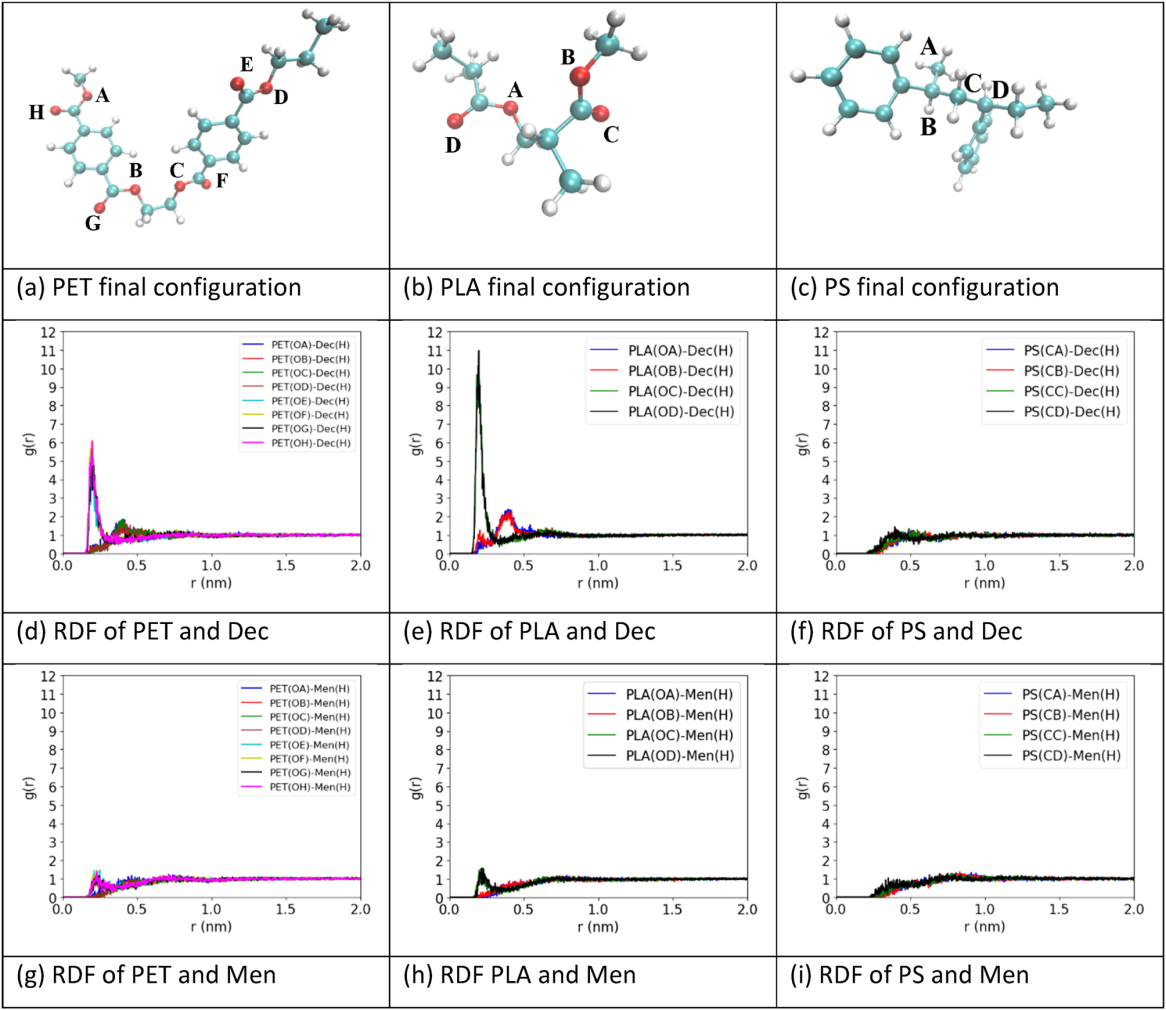
Final configuration and RDF results from the polymers in decanoic acid:menthol (1:1). (**a**–**c**) show the final configurations of PET, PLA, and PS in decanoic acid:menthol (1:1). (**d**) And (**e**) display the oxygen–hydrogen RDF between PET and PLA respectively with decanoic acid. (**f**) Displays the carbon-hydrogen RDF between PS and decanoic acid. (**g**) And (**h**) display the oxygen–hydrogen RDF between PET and PLA respectively with menthol. (**i**) Displays the carbon–hydrogen RDF between PS and menthol. The colors of the lines and legend represent the coordinating atom listed in Figure S5.

The RDFs indicate that association between decanoic acid and PLA is the most likely, with PET as the second most likely, and PS having almost no association (Fig. 5). The lack of any significant RDF peaks between the polymers and menthol also indicates that association between them is unlikely. The results from decanoic acid:menthol (1:2) are similar to decanoic acid:menthol (1:1), with no distinguishable difference in RDF for any of the polymers between the two NADES, as shown in Figure S7. The results from thymol:menthol (1:1) show smaller RDFs for PLA and PET with thymol than they did with decanoic acid, but still follow the trend of PLA having the largest, then PET, and almost no association with PS (Figure S8). The polymers had negligible RDF with menthol in this NADES as well. Figure S9 shows the RDF of the polymers in pure water solutions, with the same atoms on the polymers and the hydrogen atoms of water as the reference. The polymers showed no RDF peak, suggesting the polymers are unlikely to associate with water.

Results from the simulation systems show that there are interactions between the oxygen molecules on PLA and PET and the hydrogen molecules in decanoic acid and thymol. As RDF results explore the affinity of the plastics and NADES on a molecular level, it can help to explain the empirical extraction rate and efficiency results. The RDFs between the polymers and NADES are in general agreement with the extraction data. The strongest association was between PLA and decanoic acid, which had the highest extraction rate as well as endpoint in decanoic acid:menthol (1:1) and performed well in decanoic acid:menthol (1:2). PLA in thymol:menthol (1:1) showed significantly lower initial extraction and at the endpoint. Considering PET was able to reach an extraction percent in thymol:menthol (1:1) that was not significantly different from the decanoic acid:menthol NADES, PLA may be able to reach a higher extraction if the timeframe was extended.

PET had the second strongest association with the NADES and performed significantly lower than PLA at the two-hour extraction in both decanoic acid:menthol NADES, but not at the endpoints. In thymol:menthol (1:1), no significant difference was found between PET and PLA at the two-hour extraction point or at the endpoint. PS showed no RDF with any of the NADES and performed worse or not significantly different than the other polymers in each of the NADES at both the two-hour point and the endpoint. There was no significant difference in endpoint extractions across the polymers in each NADES which suggests that RDF is an indicator more on the extraction rate than the total percent of particles extracted. The contact angle measurements show the hydrophobicity/wettability of the plastic surface with water and NADES which is a separate factor to understanding the plastic extraction but not directly related to RDF results.

## Conclusions

This study demonstrates the potential use of NADES as extractants for removal of micro- and nano- plastic particles from an aqueous solution. The extraction rate and efficiency depend on the particle size, zeta potential, type of plastics and the interaction of NADES with the plastics. PLA was extracted at the highest rate, followed by PET, and PS. Extraction efficiency spans a range of 50–93% while the type of NADES had significant impact in the maximum percentage of polymer particles extracted. Overall, NADES made of decanoic acid:menthol (at 1:1 and 1:2 ratios) outperformed NADES made of thymol:menthol (at 1:1 ratio) especially for the extraction of PLA and PS. Molecular simulations provide insights into the affinity of plastic polymers to the NADES. PS, having the least affinity to the NADES, the smallest particle size and most negative zeta-potential, was extracted at the overall slowest rate and the lowest efficiency. Integration of experiment and computation helps to rationalize the design of hydrophobic NADES and guide the development of processes to recycle and upcycle micro- and nanoplastics from aqueous systems.

## Supplementary Information

Supplementary Information.

## Data Availability

All data generated or analyzed during this study are included in this published article and its supplementary information files.
