# Supplementary material for: Green solvent mediated extraction of micro- and nano-plastic particles from water
Source: Sci Rep. 2023 Jun 30;13:10585. doi: 10.1038/s41598-023-37490-6 (PMC10313666; doi:10.1038/s41598-023-37490-6)
Supplement: Supplementary file 1 — Supplementary Information. [file 41598_2023_37490_MOESM1_ESM.docx]

**Supporting Information**

**Green Solvent Mediated Extraction of Micro- and Nano-Plastic Particles from Water**

Jameson Hunter^1^, Yuxuan Zhang^1^, Qi Qiao^2^, Qing Shao^2^, Czarena Crofcheck^1^, Jian Shi^1, *^

Jameson R. Hunter, graduate student - Biosystems and Agricultural Engineering, 128 C.E. Barnhart Building, University of Kentucky, Lexington, KY, 40506, USA. Email: [jameson.hunter@uky.edu](mailto:jameson.hunter@uky.edu)

Qi Qiao, postdoc scholar - Chemical and Materials Engineering, 177 FPAT Building, University of Kentucky, Lexington, KY, 40506, USA. Email: [qqi227@uky.edu](mailto:qqi227@uky.edu)

Yuxuan Zhang, graduate student - Biosystems and Agricultural Engineering, 128 C.E. Barnhart Building, University of Kentucky, Lexington, KY, 40506, USA. Email: [yx.zhang@uky.edu](mailto:yx.zhang@uky.edu)

Qing Shao, Assistant Professor - Chemical and Materials Engineering, 177 FPAT Building, University of Kentucky, Lexington, KY, 40506, USA. Email: [qshao@uky.edu](mailto:qshao@uky.edu)

Czarena Crofcheck, Professor - Biosystems and Agricultural Engineering, 128 C.E. Barnhart Building, University of Kentucky, Lexington, KY, 40506, USA. Email: [crofcheck@uky.edu](mailto:crofcheck@uky.edu)

Jian Shi, Associate Professor - Biosystems and Agricultural Engineering, 128 C.E. Barnhart Building, University of Kentucky, Lexington, KY, 40506, USA. Email: [j.shi@uky.edu](mailto:j.shi@uky.edu)

^*^Corresponding to: Dr. Jian Shi ([j.shi@uky.edu](mailto:j.shi@uky.edu)) University of Kentucky

Phone: (859) 218-4321; Fax: (859) 257-5671

**Number of pages: 13**

**Number of tables: 2**

**Number of figures: 9**


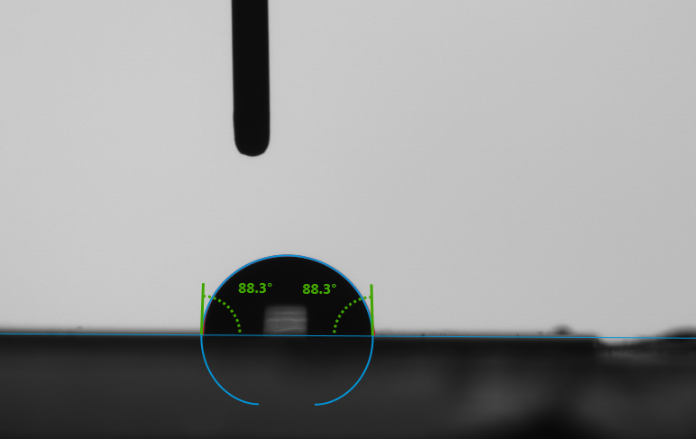

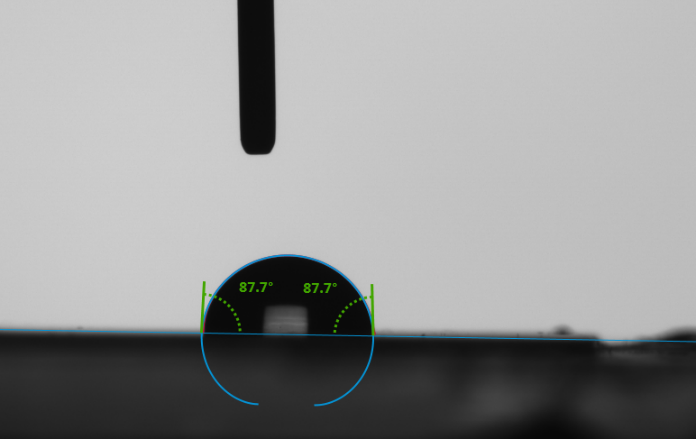

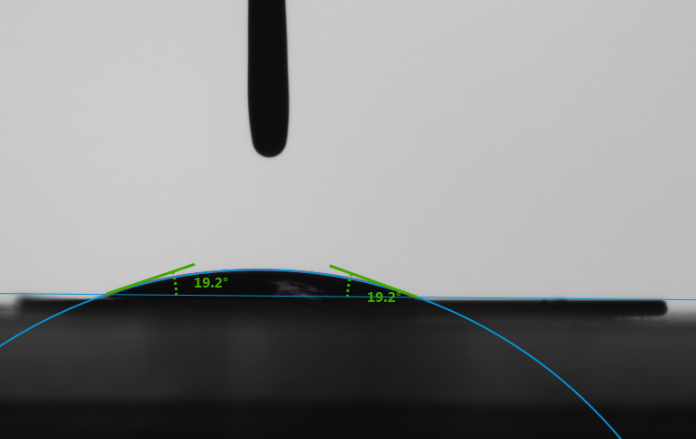

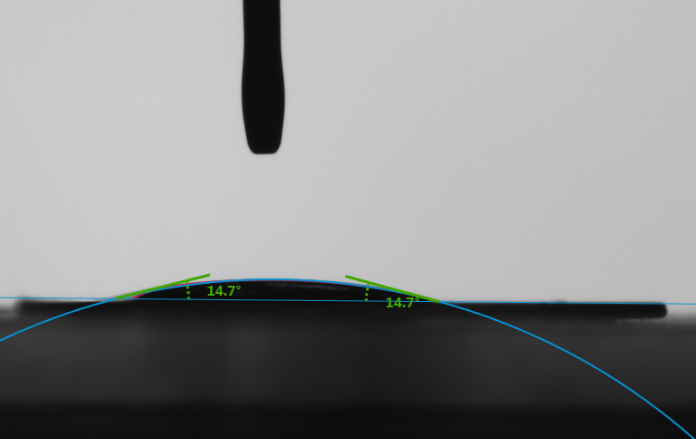

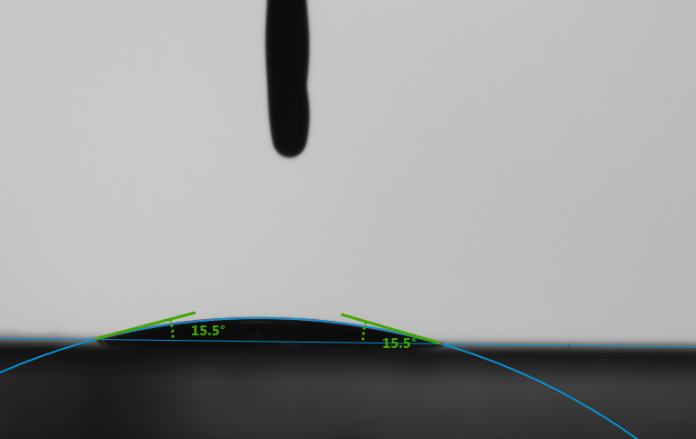

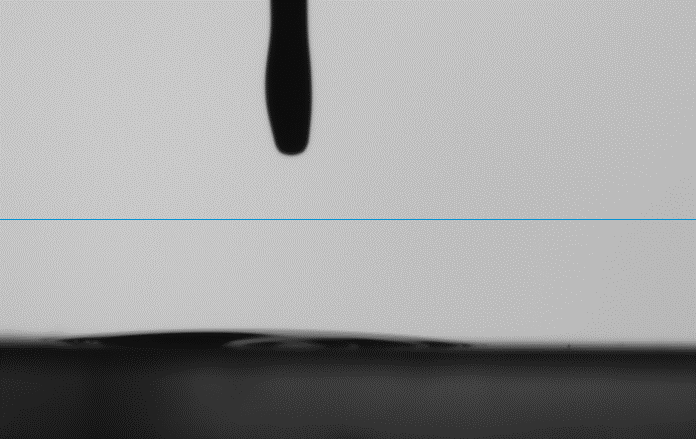

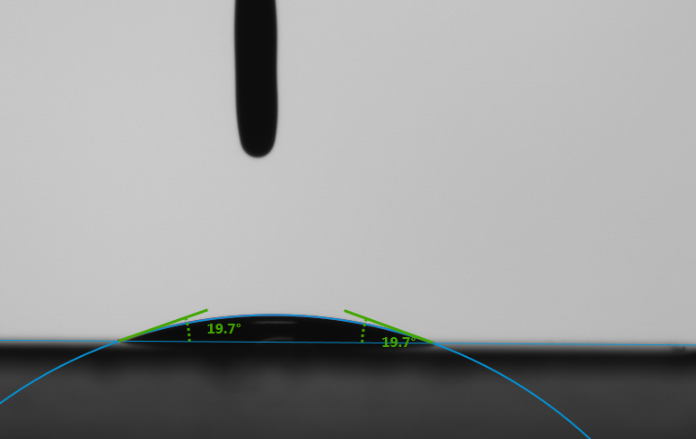

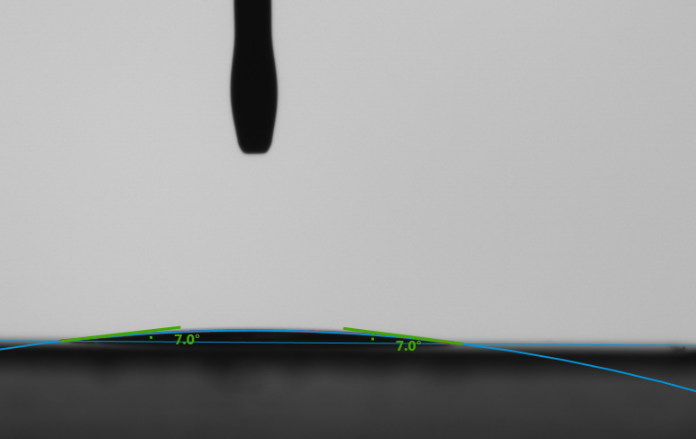


**(a)**

**(b)**

**(c)**

**(d)**

**Figure S1** Contact angles of **a)** water, **b)** decanoic acid:menthol (1:1), **c)** decanoic acid:menthol (1:2), and **d)** thymol:menthol (1:1) at 0 seconds (left) and 30 seconds (right) on PS surface.


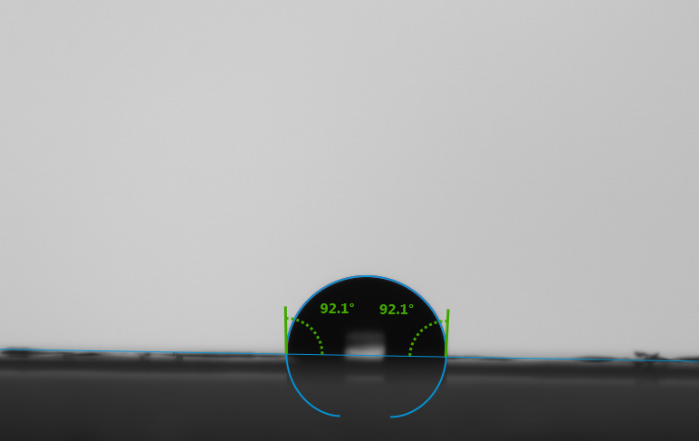

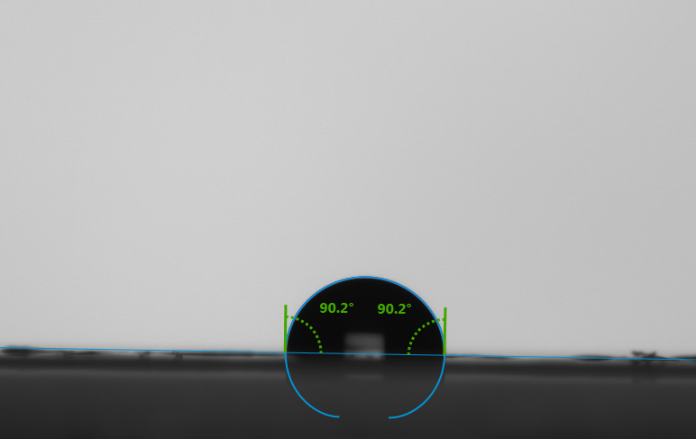

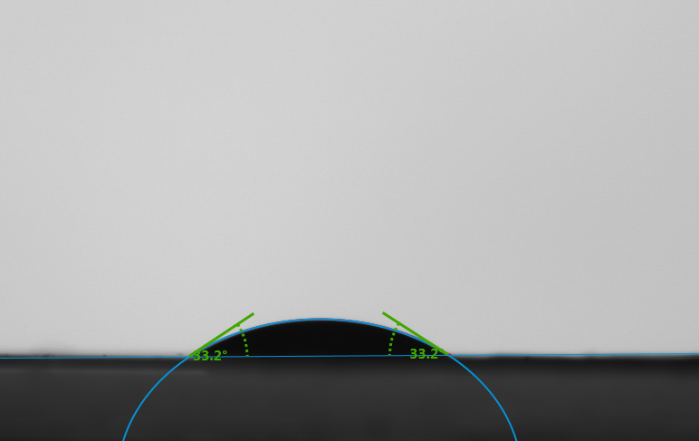

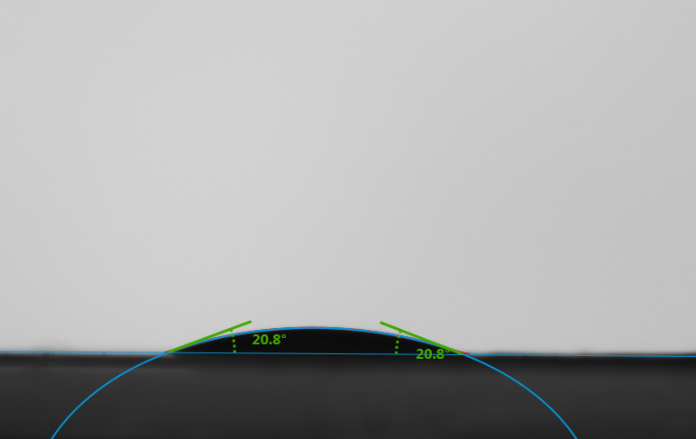

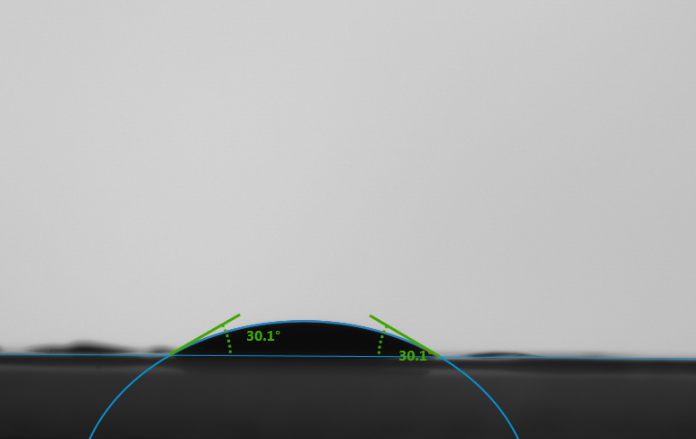

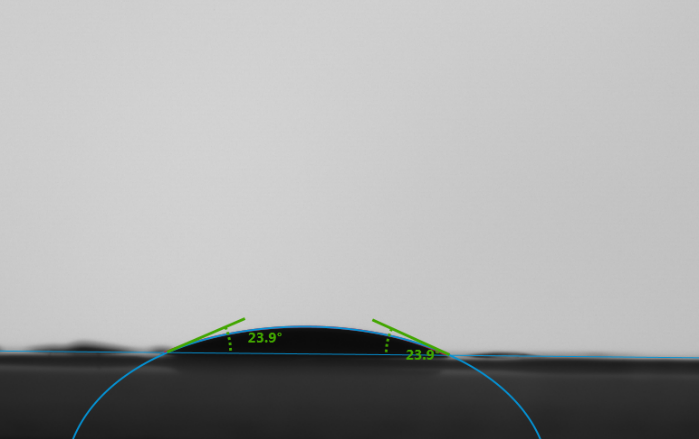

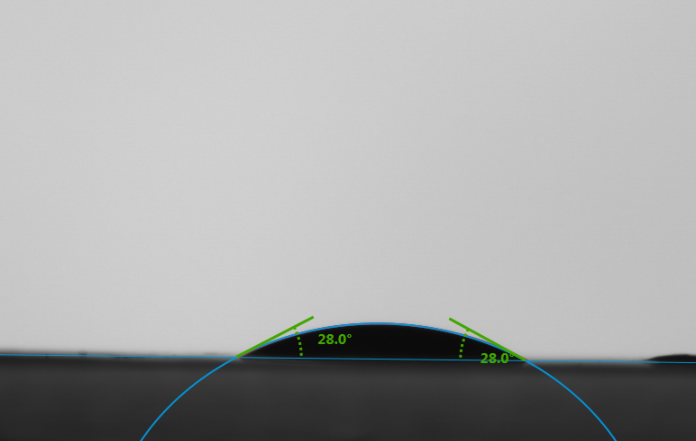

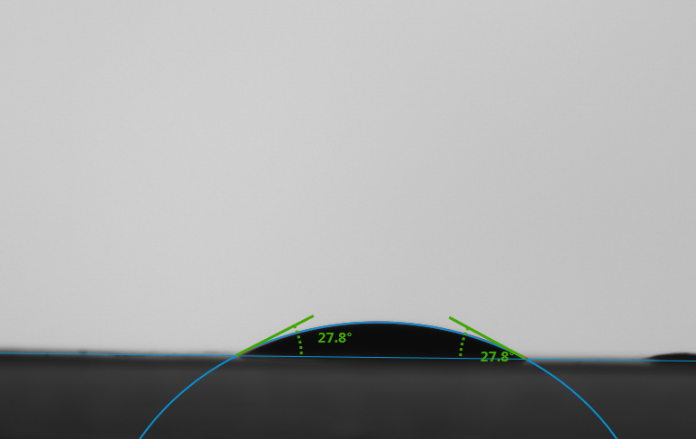


**(a)**

**(b)**

**(c)**

**(d)**

**Figure S2** Contact angles of **a)** water, **b)** decanoic acid:menthol (1:1), **c)** decanoic acid:menthol (1:2), and **d)** thymol:menthol (1:1) at 0 seconds (left) and 30 seconds (right) on PLA surface.

**
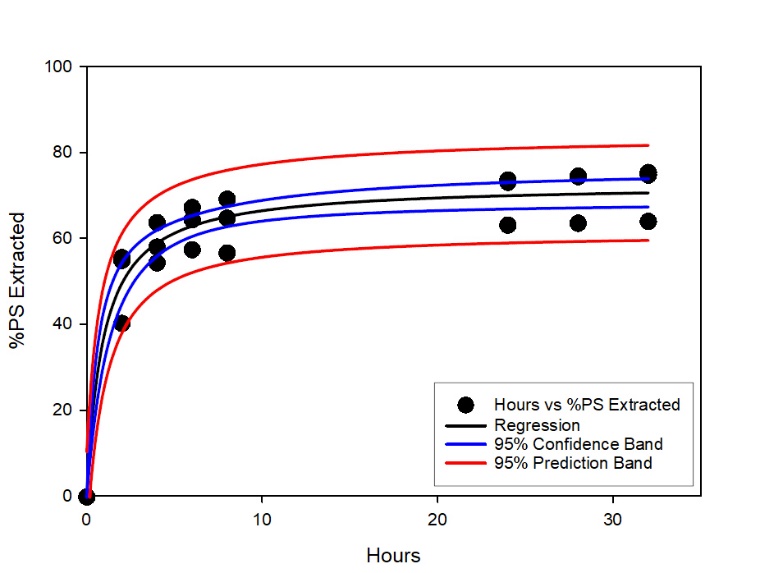

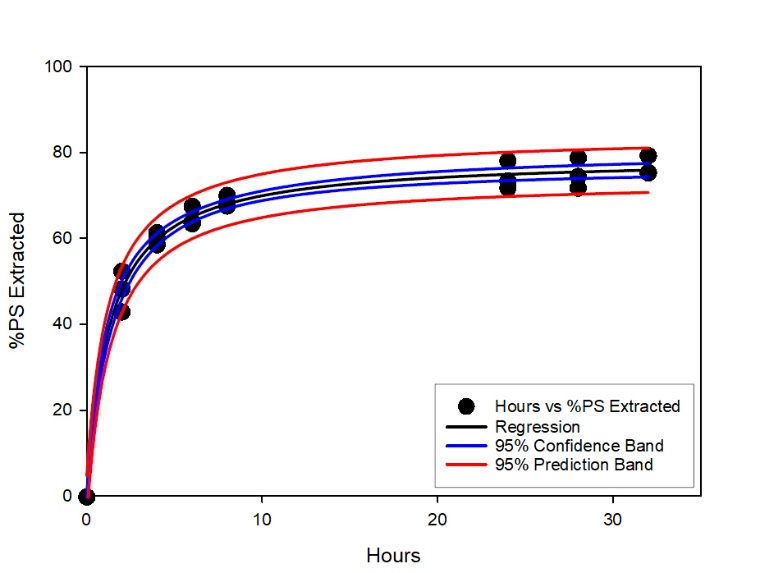

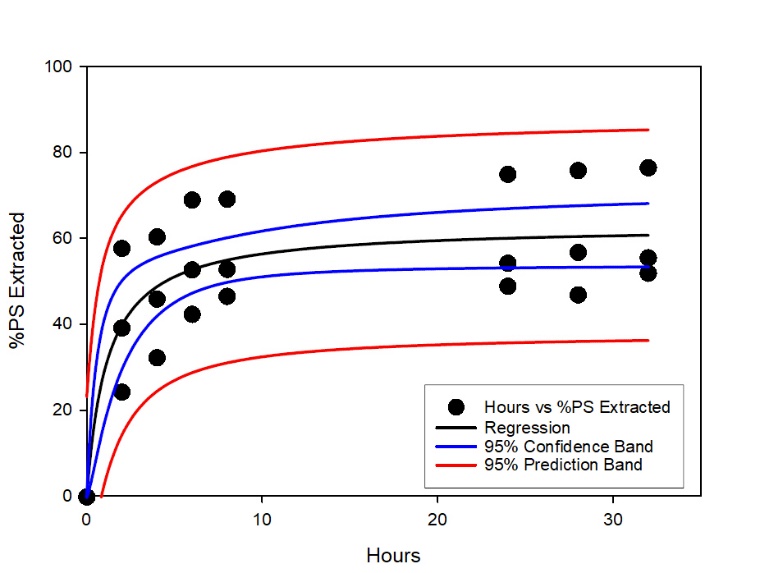
**

**Figure S3** Percent of PS extracted by three types of NADES: **a)** Decanoic Acid:Menthol (1:1);

**b)** Decanoic Acid:Menthol (1:2); and **c)** Thymol:Menthol (1:1).

**Figure S4** Percent of PLA extracted by three types of NADES: **a)** Decanoic Acid:Menthol (1:1);

**b)** Decanoic Acid:Menthol (1:2); and **c)** Thymol:Menthol (1:1).

***Molecular Interaction Simulations***

The all-atom model was used to describe the polyethylene terephthalate (PET), polylactic acid (PLA), polystyrene (PS), decanoic acid (Dec), menthol (Men), and thymol molecules, while the TIP 4P model (Jorgensen, Chandrasekhar et al. 1983) was used for the water molecules. Figure S5 shows the structures of the six molecules.

| 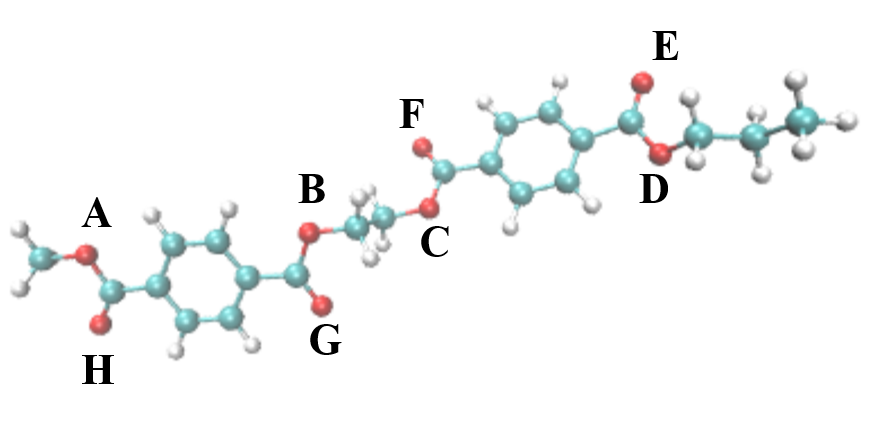 | 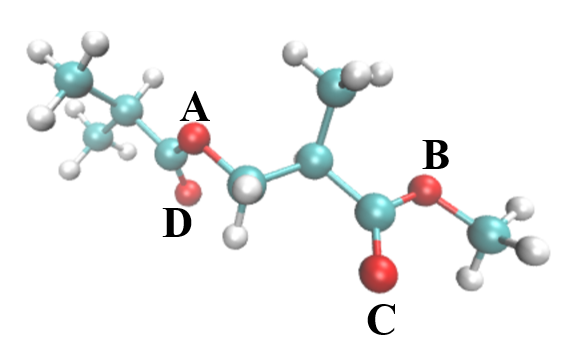 | 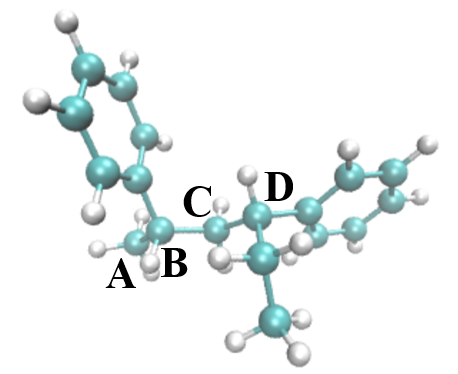 |
| --- | --- | --- |
| (a) PET | (b) PLA | (c) PS |
| 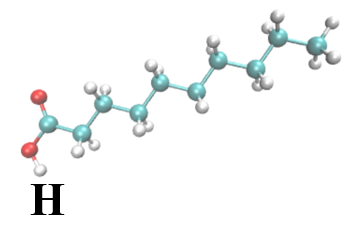 | 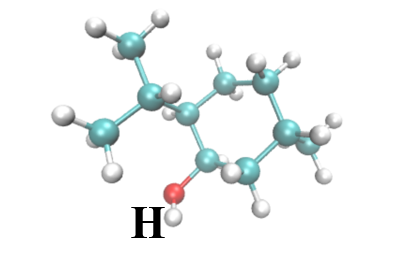 | 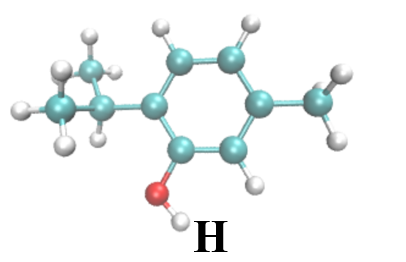 |
| (d) Decanoic acid | (e) Menthol | (f) Thymol |

**Figure S5** Molecular structures of the three plastics: (a) PET, (b) PLA, (c) PS, and three organic molecules formed the three NADESs: (d) decanoic acid, (e) menthol and (f) thymol. The molecules are shown in the CPK model (C: cyan, O: red, and H: white). All the oxygen atoms on PET and PLA, four carbon atoms on the mainchain of PS, and all hydrogen atoms on the hydroxyl groups of the three organic molecules are labelled. These labels were used to calculate RDF.

The nonbonded and bonded interactions in the system were described using the OPLSAA/M force field (Robertson, Tirado-Rives et al. 2015) because this force field can properly describe the behavior of organic molecules. The force field parameters were assigned using the Ligpargen web server (William L. Jorgensen* 2005, Dodda, Cabeza de Vaca et al. 2017, Dodda, Vilseck et al. 2017).


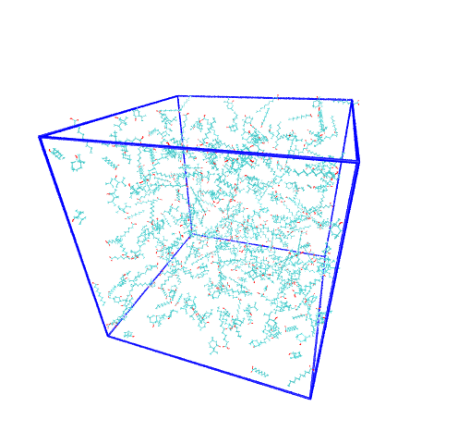


**Figure S6** Snapshot of the initial configuration of PET in Dec-Men11 containing 1 PET, 200 Dec, and 200 Men molecules. Color representations are same as in Figure S5.

The simulation systems of nine plastic-NADESs were created by placing a plastic molecule in a cubic box and filling the box with specific numbers of solvent molecules. The creation of the simulation box was fulfilled using the insert-molecule and solvate tools of GROMACS (Abraham, Murtola et al. 2015). Table S1 shows the details of the twelve NADES and water systems. Figure S6 shows the snapshot of PET in De*c*-Men11.

**Table S1** Components, molar ratio, and numbers of molecules in the nine plastic-NADES systems.

| System label | Component A | Component B | NADES molar ratio | Total number of solvent molecules |
| --- | --- | --- | --- | --- |
| PET-Dec-Men11 | Decanoic Acid | Menthol | 1:1 | 400 |
| PET-Dec-Men12 | Decanoic Acid | Menthol | 1:2 | 600 |
| PET-Thy-Men11 | Thymol | Menthol | 1:1 | 400 |
| PET-water | water | - | - | 4115 |
| PLA-Dec-Men11 | Decanoic Acid | Menthol | 1:1 | 400 |
| PLA-Dec-Men12 | Decanoic Acid | Menthol | 1:2 | 600 |
| PLA-Thy-Men11 | Thymol | Menthol | 1:1 | 400 |
| PLA-water | water | - | - | 4128 |
| PS-Dec-Men11 | Decanoic Acid | Menthol | 1:1 | 400 |
| PS-Dec-Men12 | Decanoic Acid | Menthol | 1:2 | 600 |
| PS-Thy-Men11 | Thymol | Menthol | 1:1 | 400 |
| PS-water | water | - | - | 3590 |

This work deploys the OPLSAA/M force field (Robertson, Tirado-Rives et al. 2015) to describe bonded and nonbonded interactions in the systems. The OPLSAA/M force field has been widely used for simulating small molecules and biomolecules. The non-boned interactions are a sum of short-range Lennard-Jones 12-6 potential and long-range coulombic potential, as shown in Equation 1. The bonded interactions are a sum of the bond, angle, and dihedral potentials, as described in the force field.

$E_{ij}\left( r_{ij} \right)=4\varepsilon_{ij}\left( \left( \frac{\sigma_{ij}}{r_{ij}} \right)^{12}-\left( \frac{\sigma_{ij}}{r_{ij}} \right)^{6} \right)+\frac{e_{i}e_{j}}{4\pi\varepsilon_{0}r_{ij}}$ (1)

where $E_{ij}$ is the potential energy due to the nonbonded interactions between atoms i and j, $r_{ij}$ is the distance between atoms *i* and *j*, $\varepsilon_{ij}$ is the energetic parameter, $\sigma_{ij}$ is the geometric parameter and $e_{i}$ is the partial charge of atom *i*. The Jorgensen mixing rule is applied to obtain $\varepsilon_{ij}$ and $\sigma_{ij}$ for atoms belonging to different types.

A three-step simulation process is conducted for every simulation system. First, energy minimization was conducted to remove any too-close contacts between atoms. Second, three simulations, a 50-ns isobaric-isothermal (NPT, T=373 K, P= 100 KPa), a 50-ns isobaric-isothermal (NPT, T=335 K, P= 100 KPa), and a 50-ns isobaric-isothermal (NPT, T=298 K, P= 100 KPa) ensemble MD simulation (integral step = 2 fs) were conducted to let the system reach thermodynamic equilibrium. Third, a 1000-ns isobaric-isothermal (NPT, T=298 K) ensemble MD simulation (integral step = 2 fs) was conducted to collect the trajectory at a frequency of 50 ps. The Berendsen method(Berendsen, Postma et al. 1984) is used to control the temperature and pressure of the system in the second step because it allows the system to reach the desired pressure and temperature at a fast pace. The Parrinello-Rahman method (Parrinello and Rahman 1981) is used to control the pressure of the system in the third step to collect the mean square displacement (MSD). The velocity-rescaling method (Bussi, Donadio et al. 2007) is used to control the temperature of the system in the third step. The short-range van der Waals interactions use a 1.2-nm cut-off, and the long-range electrostatic interactions were calculated using the particle mesh Ewald sum.(Darden, York et al. 1993) All bonds involving H atoms were constrained during the simulations. The energy minimization and MD simulations for all the systems were conducted using Gromacs-2021 (Esquembre, Sanz et al. 2013).

| 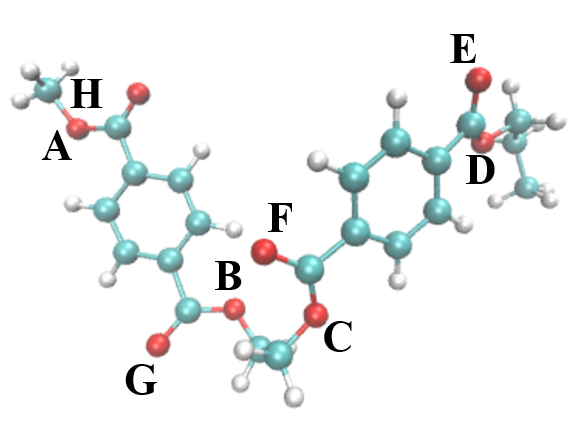 | 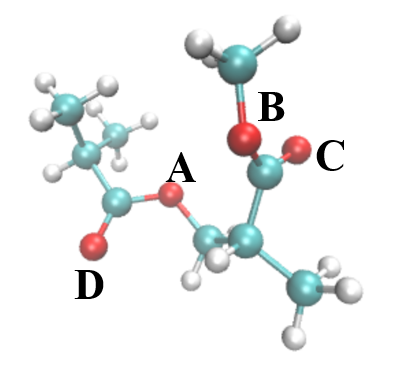 | 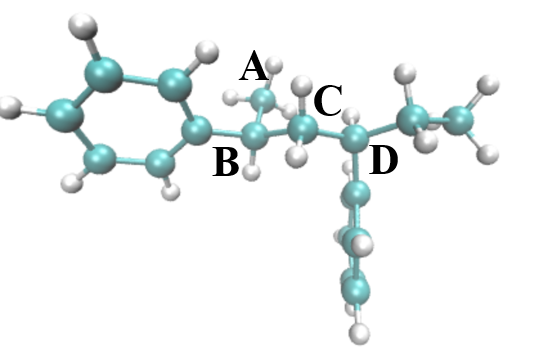 |
| --- | --- | --- |
| (a) PET final configuration | (b) PLA final configuration | (c) PS final configuration |
| 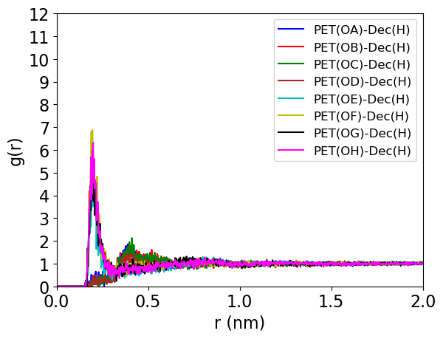 | 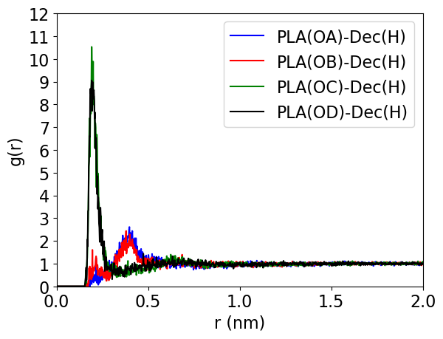 | 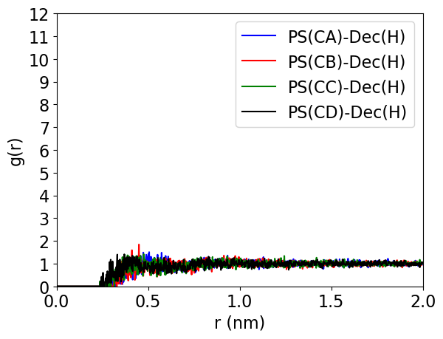 |
| (d) RDF of PET and Dec | (e) RDF of PLA and Dec | (f) RDF of PS and Dec |
| 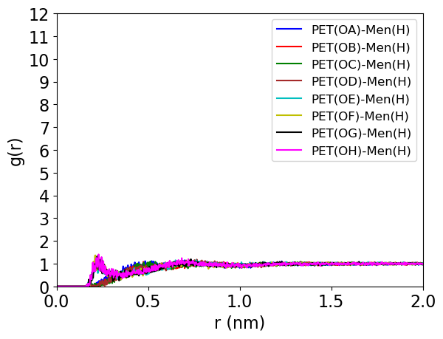 | 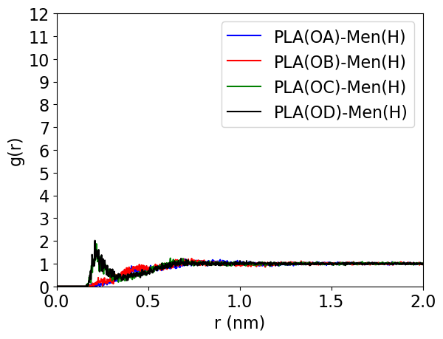 | 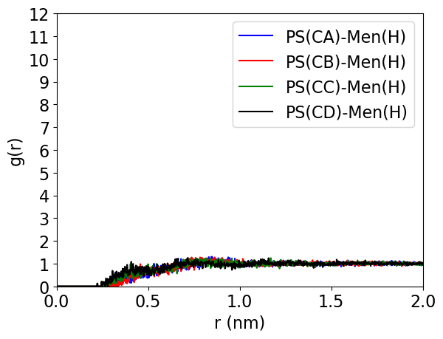 |
| (g) RDF of PET and Men | (h) RDF PLA and Men | (i) RDF of PS and Men |

**Figure S7** Final configuration and RDF results from the polymers in decanoic acid:menthol (1:2). (a)-(c) show the final configurations of PET, PLA, and PS. (d) and (e) display the oxygen-hydrogen RDF between PET and PLA respectively with decanoic acid. (f) displays the carbon-hydrogen RDF between PS and decanoic acid. (g) and (h) display the oxygen-hydrogen RDF between PET and PLA respectively with menthol. (i) displays the carbon-hydrogen RDF between PS and menthol.

| 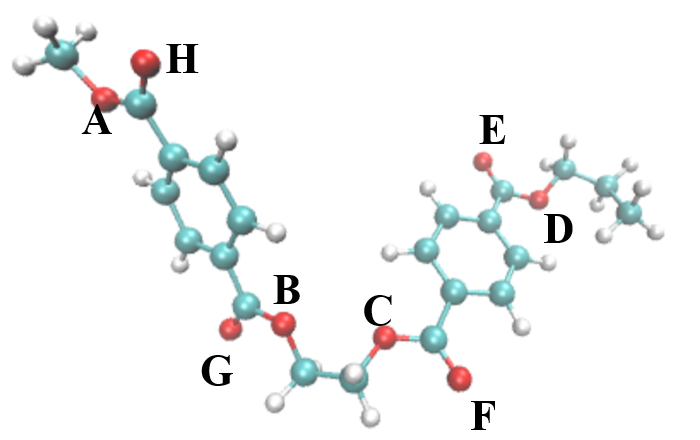 | 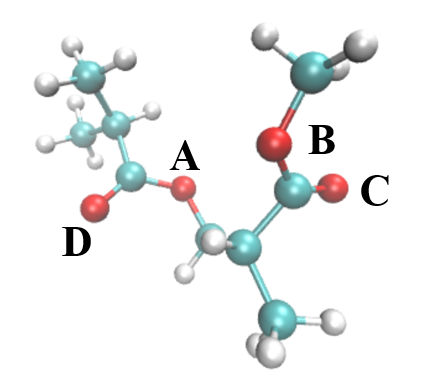 | 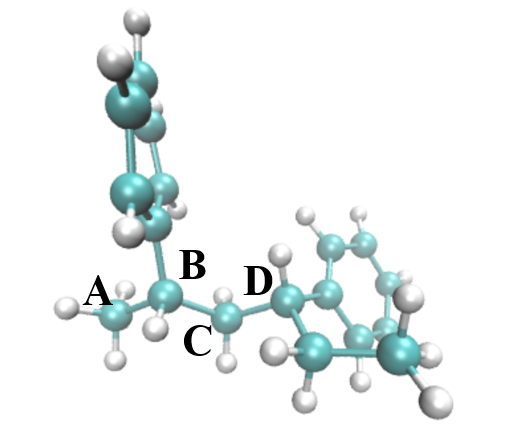 |
| --- | --- | --- |
| (a) PET final configuration | (b) PLA final configuration | (c) PS final configuration |
| 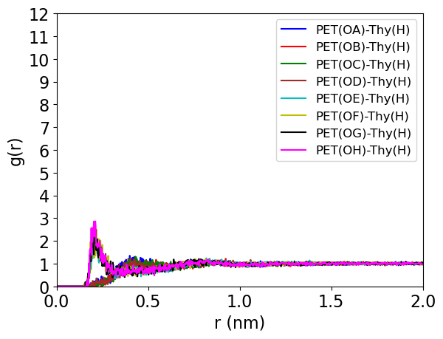 | 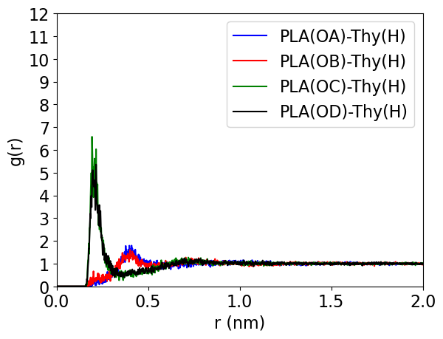 | 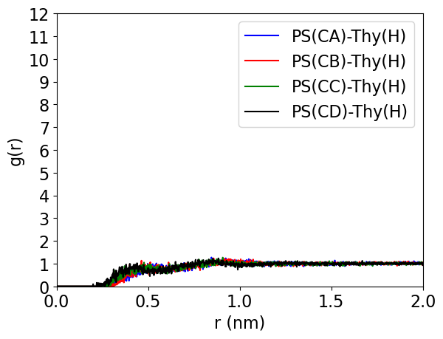 |
| (d) RDF of PET and Thy | (e) RDF of PLA and Thy | (f) RDF of PS and Thy |
| 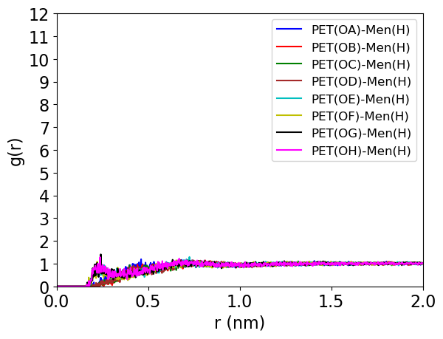 | 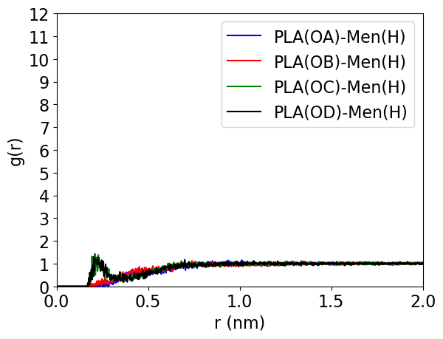 | 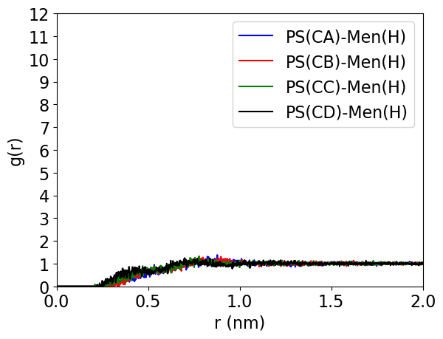 |
| (g) RDF of PET and Men | (h) RDF PLA and Men | (i) RDF of PS and Men |

**Figure S8** Final configuration and RDF results from the polymers in thymol:menthol (1:1). (a)-(c) show the final configurations of PET, PLA, and PS. (d) and (e) display the oxygen-hydrogen RDF between PET and PLA respectively with thymol. (f) displays the carbon-hydrogen RDF between PS and thymol. (g) and (h) display the oxygen-hydrogen RDF between PET and PLA respectively with menthol. (i) displays the carbon-hydrogen RDF between PS and menthol.

| 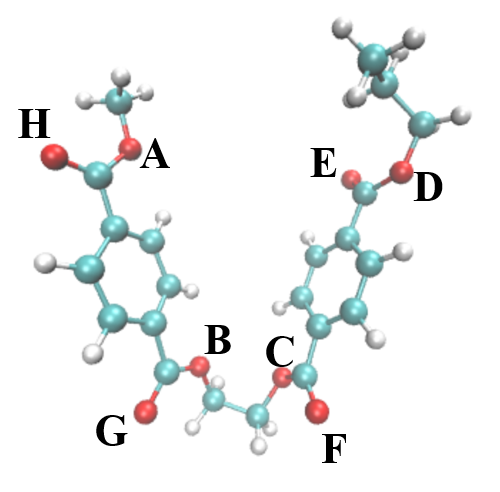 | 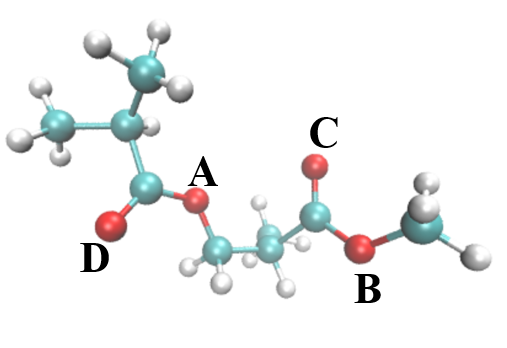 | 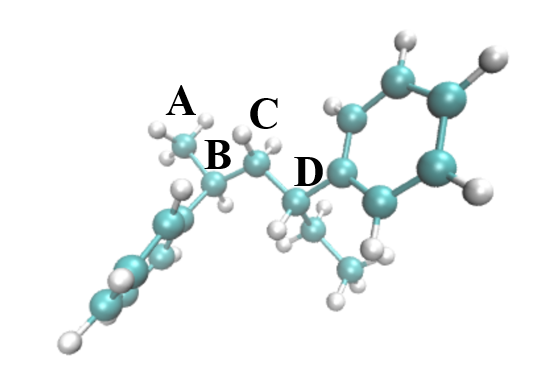 |
| --- | --- | --- |
| (a) PET final configuration | (b) PLA final configuration | (c) PS final configuration |
| 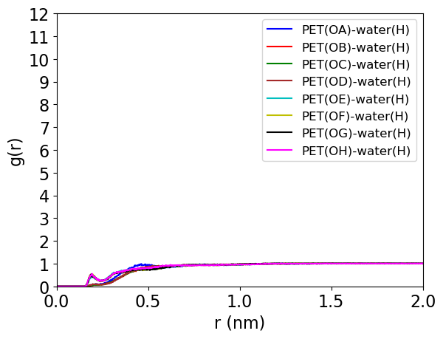 | 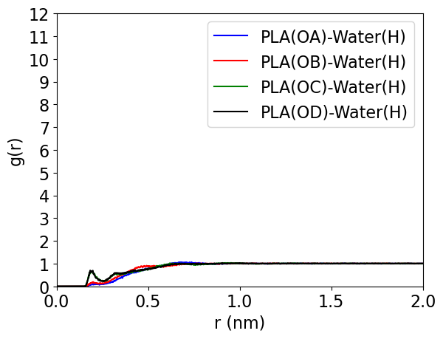 | 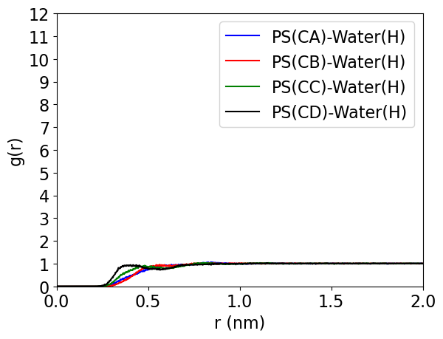 |
| (d) RDF of PET and water | (e) RDF of PLA and water | (f) RDF of PS and water |

**Figure S9** Final configuration and RDF results from the polymers in water. (a)-(c) show the final configurations of PET, PLA, and PS. (d) and (e) display the oxygen-hydrogen RDF between PET and PLA respectively with water. (f) displays the carbon-hydrogen RDF between PS and water.

***Diffusion coefficient***

The diffusion coefficient (De) is the average value of four 500-ns MD simulations. It indicates how fast the plastic could move in an equilibrium DES or water solution. Table two shows that plastic-water systems show the highest De since water has the lowest viscosity. The Thy-Men11 DES shows higher De than the other two DESs for all three plastics. The PLA could move faster in the DESs and water solution compared with the other two plastics.

**Table S2** The diffusion coefficient of three plastics in the DESs and water solutions.

|  | PET | PLA | PS |
| --- | --- | --- | --- |
|  | De (10-5 cm^2^/s) | De (10-5 cm^2^/s) | De (10-5 cm^2^/s) |
| Dec-Men11 | 0.15±0.046 | 0.18±0.012 | 0.18±0.066* |
| Dec-Men12 | 0.20±0.054 | 0.22±0.035 | 0.20±0.077* |
| Thy-Men11 | 0.22±0.152* | 0.29±0.081 | 0.24±0.063* |
| Water | 0.66+0.184 | 1.00±0.427 | 0.78±0.085* |

* The data is collected shorter than 500ns.
